# Tilting the lasso by knowledge-based post-processing

**DOI:** 10.1186/s12859-016-1210-7

**Published:** 2016-09-02

**Authors:** Kukatharmini Tharmaratnam, Matthew Sperrin, Thomas Jaki, Sjur Reppe, Arnoldo Frigessi

**Affiliations:** 1 Department of Mathematics and Statistics, Lancaster University, Lancaster, UK; 2 Institute of Population Health, The University of Manchester, Manchester, UK; 3 Department of Medical Biochemistry, Oslo University Hospital, Oslo, Norway; 4 Lovisenberg Diakonale Hospital, Oslo, Norway; 5 Institute of Basic Medical Sciences, University of Oslo, Oslo, Norway; 6 Oslo Centre for Biostatistics and Epidemiology, University of Oslo and Oslo University Hospital, Oslo, Norway

**Keywords:** Bone mineral density, Elicitation, Lasso

## Abstract

**Background:**

It is useful to incorporate biological knowledge on the role of genetic determinants in predicting an outcome. It is, however, not always feasible to fully elicit this information when the number of determinants is large. We present an approach to overcome this difficulty. First, using half of the available data, a shortlist of potentially interesting determinants are generated. Second, binary indications of biological importance are elicited for this much smaller number of determinants. Third, an analysis is carried out on this shortlist using the second half of the data.

**Results:**

We show through simulations that, compared with adaptive lasso, this approach leads to models containing more biologically relevant variables, while the prediction mean squared error (PMSE) is comparable or even reduced. We also apply our approach to bone mineral density data, and again final models contain more biologically relevant variables and have reduced PMSEs.

**Conclusion:**

Our method leads to comparable or improved predictive performance, and models with greater face validity and interpretability with feasible incorporation of biological knowledge into predictive models.

**Electronic supplementary material:**

The online version of this article (doi:10.1186/s12859-016-1210-7) contains supplementary material, which is available to authorized users.

## Background

In medicine and genetics it is common to have a large number of covariates available to predict a response. Prior information concerning the relevance of each covariate in predicting the response may also be available. The response could be, for example, bone mineral density (BMD), and the covariates are expression levels of 20,000 genes, measured in 100 independent samples. Other examples include disease subtyping, as in breast cancer where the aim is to predict the type of disease (by multinomial regression say) based on gene expressions. There are two challenges when modelling such data. First, the number of covariates, *P*, is often much larger than the number of samples, *n*. Second, it may not be feasible, or practical, to elicit prior information concerning such a large number of covariates, *P*.

The first challenge, dealing with *P*>*n*, has been thoroughly studied, particularly over the last two decades. When fitting regression models, instability and overfitting problems can be mitigated using regularization methods, which shrink the coefficients of the regression model towards zero; the extent of shrinkage is controlled by a regularization parameter, which is commonly chosen to minimize the out-of-sample prediction error.

One commonly studied regularization method is the lasso [1], which adds a *L*_1_ penalty to the likelihood that relates the predictors to the response. The regularization parameter is typically chosen by cross validation. The lasso has the appealing property that it induces sparseness: in the selected model, generally only a small number of covariates have a non-zero coefficient. In many fields, such as genetics, it is reasonable to believe that this corresponds to reality — with only a small subset of the available genetic markers being needed to predict a phenotype. Accurate recovery of a subset of relevant independent predictors is of interest both to understand underlying aetiological processes, and to produce a predictive model that is transparent, has face validity and has good generalizability.

There are results describing the settings in which the lasso can recover the correct covariates, and ignore the incorrect ones [2–4]. However, these results are asymptotic, consequently this property is not guaranteed in finite samples, and place strong restrictions on the covariate design matrix — typically related to ensuring that the covariates are not too highly correlated with each other and that the signal (in terms of the absolute value of the regression coefficients of the true variables) strongly exceeds the noise, i.e. the absolute value of the coefficients of the nuisance variables. In practice, sample sizes are small, covariates are highly correlated, and signal to noise ratios may be low, so we cannot expect to recover the correct set of covariates with a naive use of lasso.

Various extensions of lasso have therefore been proposed that partially address these issues. For example, adaptive lasso [3] allows the *L*_1_ penalty to apply unequally to different coefficients, and in doing so has improved performance compared with lasso in recovering the correct predictors. The elastic net [5] tends to include clusters of correlated variables, whereas lasso would typically include a single representative, so elastic net has better sensitivity for detecting relevant predictors, at the expense of poorer specificity. Besides the different approaches to select relevant covariates, a number of different ideas have been presented that allow the relevance of the selected covariates to be inferred [6–8].

The second challenge, feasibly eliciting prior information for a large number of covariates, is the primary focus of this paper. In genetics, for example, many genes are already believed or known to play important roles in the underlying mechanistic processes related to a phenotypic outcome. The generality and reliability of such understanding varies, with certain genes clearly playing a strong role in diseases (like P53 in cancer, e.g [9]), but the role of other genes being more hypothetical. Biologists and clinicians would be rightly skeptical of the value of a predictive model, if none of the well understood trait-relevant genes were present. Therefore, a balance must be struck between the face validity and generalizability of the model, through inclusion of a prior that favours trait-relevant genes. In principle, this can be achieved in a Bayesian framework, which favours data with prior biological knowledge. This can be combined with the lasso using Bayesian lasso [10], or the weighted lasso [11].

However, to use a Bayesian approach, an expert such as a geneticist or biologist would be required to produce a prior distribution over a large number of variables (in excess of 1000 variables, even after pre-screening). This is infeasible both because of the size of the task, and mostly because of the difficulty of formalizing prior knowledge for predicting a specific trait, based on knowledge about molecular processes known to influence related traits. For example, a limited number of genes are known to play a role in bone formation, and variation in bone mineral density (BMD) but it is likely that many so far unrecognized genes are also important for BMD. Therefore, construction of appropriate informative priors could be guided by also having some information available about the predictive ability of the genes for the outcome under consideration.

In this paper, we present an approach that allows biological expectations on the role of a reasonable number of genes in prediction of a specific outcome to be elicited, and incorporated into a model, in a manageable and coherent manner. This is made possible through a two-stage approach. In the first step, we use the data only to determine, by cross-validation guided adaptive lasso, the optimal set for prediction of the phenotype. For each gene in the adaptive lasso set, we derive a further set of genes (denoted a *bag* of genes) that could take the place of the gene in the adaptive lasso set with minimal loss in predictive power. The union over all bags of genes, which will be substantially smaller than the initial longlist of genes, is then taken forward. Biologists are then asked to assess the biological importance for each gene in the bag union.

This knowledge can be expressed in various ways, including full ranking of all genes in each bag, in terms of expected biological relevance. To simplify, we assume here that genes are simply classified as biologically relevant or not for the trait under investigation. A gene in the lasso set can then be switched with a biologically relevant gene present in its bag. The final model is fitted using separate data to the first step. This method allows us to find gene sets with predictive power, which have been derived from relevant data sets, but incorporating biological knowledge in a feasible manner. We show through simulations that this contributes to robust prediction signatures.

## Methods

Consider a linear model

1 $$ E\left[Y_{i}\right] = \sum_{j=1}^{P} \beta_{j} X_{ij},~i=1,2,\ldots,n,  $$

where *Y*_*i*_ is a response variable (centered to have zero mean) measured in sample *i*, *X*_*ij*_ is the *j*^*th*^ covariate measured in sample *i* (centered to have zero mean, and scaled to have unit variance), *i*=1,…,*n* and *j*=1,…,*P* with potentially *P*≫*n*. Here, we used centered mean and unit variance for easy to interpret the regression models. The response may be a continuous phenotype and the covariates may be genes.

We wish to build a sparse model that incorporates biological information on the relevance of the covariates, to produce a final model with a relevant aetiological interpretation. We do so via a two-stage approach. First the data are split into three portions: $\mathcal {D}_{1} = \left \{\left (Y_{i},X_{i1},\ldots,X_{iP}\right)\!;~\! i\,=\,1,\ldots,m\right \}$, $\mathcal {D}_{2} = \left \{(Y_{i},X_{i1},\ldots,X_{iP}); i = m+1, \ldots, 2m\right \}$ and $\mathcal {D}_{3} = \left \{(Y_{i},X_{i1},\ldots,X_{iP}); i = 2m+1, \ldots, n\right \}$ where *m*=*n*/3 is our default choice. Stage one of the approach uses $\mathcal {D}_{1}$ to produce a shortlist of covariates that are potential members of the final sparse model; we then elicit for each of these covariates whether it is biologically relevant or not. Stage two uses $\mathcal {D}_{2}$ to fit the final sparse model, incorporating this relevance information. The final third ($\mathcal {D}_{3}$) is only necessary in our simulation experiments, where it is used for validation of the model derived in the second stage. In real applications the data can be divided into two portions.

### Bags of biologically relevant variables

The lasso [1] involves an optimisation of the form

2 $$ \hat{\boldsymbol{\beta}}_{\text{lasso}} = {\underset{\boldsymbol{\beta}}{\text{argmin}}}\left\|\boldsymbol{Y} -\boldsymbol{X}\boldsymbol{\beta}\right\|_{2}^{2} + \lambda \left\|\boldsymbol{\beta}\right\|_{1},  $$

which is the usual least squares minimisation plus an *L*_1_ penalty on the *β*s, where *λ* is the tuning parameter denoting the severity of the penalty. An equivalent formulation is also available for generalised linear models and Cox proportional hazard models. All of these can be solved efficiently using the *glmnet* package in *R* [12].

The adaptive lasso [3] modifies the lasso by introducing weights

3 $$ \hat{\boldsymbol{\beta}}_{\text{lasso}} = {\underset{\boldsymbol{\beta}}{\text{argmin}}}\| \boldsymbol{Y} -\boldsymbol{X\beta}\|_{2}^{2} + \lambda \Vert \boldsymbol{w}\boldsymbol{\beta} \|_{1},  $$

where the weight vector ***w*** comprises the reciprocal absolute values of the ordinary least squares coefficients. This modification means the adaptive lasso consistently estimates variables, while the original lasso does not [3].

The first step of the proposed approach is to fit an adaptive lasso model, using data $\mathcal {D}_{1}$, to select a list of covariates, denoted by *S*, where *k*-fold cross validation is used to determine the penalty *λ*.

Next we propose a range of approaches to augment the list of covariates *S*, selected by the adaptive lasso. The general idea is that for each covariate *j*∈*S*, we generate a *bag* of covariates that are potential substitutes. Covariates may appear in more than one bag. Three different methods for selecting the bags of covariates are considered:

- Bag type 1 (B1):
  For each *j*∈*S*, compute the correlation Corr(*X*_*j*_,*X*_*k*_) for all other covariates *k*≠*j*. Take the *q* covariates that are correlated with each selected covariate from adaptive lasso most. This produces a bag of alternative covariates for each gene in the adaptive lasso set.
- Bag type 2 (B2):
  Instead of using a fixed bag size, define a threshold on the correlation and select covariate *X*_*j*_ if its correlation exceeds this threshold; e.g. select covariate *X*_*k*_ if Corr(*X*_*j*_,*X*_*k*_)≥0.25.
- Bag type 3 (B3):
  For each *j*∈*S*, compute the mean squared error (MSE) when replacing *X*_*j*_ with *X*_*k*_, for each *j*≠*k*, using ordinary least squares in the regression with |*S*| covariates. Usually |*S*|<*n*. Take the *q* covariates with the smallest MSE.

We assume that every covariate in the bag can be a priori classified as preferred or not, from a subject matter biological point of view. We call the preferred variables *biologically relevant variables*. Given the bags, the next step is to consider switching each variable selected by lasso with a variable contained in the corresponding bag.

- If there is just one biologically relevant covariate in the bag, then we switch it with the corresponding covariate selected by adaptive lasso.
- If we find more than one biologically relevant covariate in the same bag, we need to select which one to switch with the corresponding originally selected one. For bag of type B1 and type B2 we chose the biologically relevant covariate which has the largest correlation with the one selected by adaptive lasso. For bag of type B3 we chose the covariate which leads to the small prediction mean square error when switched with the one selected by adaptive lasso.
- If the lasso selects a biologically relevant variable, it is kept.
- If there are no biologically relevant covariates in the bag, then the original covariate from the adaptive lasso solution is retained.

In bag type B3, if for a bag variable the MSE ratio (MSE of replaced model/MSE of adaptive lasso model) is less than one, then introducing that variable is particularly appealing because this leads to a reduction in the MSE of the model. Note that should a biologically relevant variable be selected into the model from two or more bags, it will appear only once in the final model (thus leading to a sparser final model).

For comparison, we also present results using the standard lasso in the first step. All fitting is carried out efficiently using the *glmnet* package in *R* [12].

## Results

### Simulation experiments

We test the performance of the proposed approach compared with a standard lasso approach, using simulations. We take the sample size *n*=300 and the number of covariates *P*=1000. The true model which is used to generate the outcome has 20 covariates *x*_*j*_,*j*=1,…,20:

4 $$ y_{i}= \sum_{j=1}^{20}\beta_{j} x_{ij}+ \epsilon_{i},  $$

where *ε*_*i*_∼*N*(0,*σ*^2^), with *σ*^2^ chosen to achieve a range of signal to noise ratios (SNR). Different *β*_*j*_ values are also chosen, across different simulation settings. Additional biologically relevant covariates *x*_*j*_,*j*=21,…,60, were generated with varying levels of correlation with the true covariates *x*_*j*_,*j*=1,…,20. The true covariates are themselves considered biologically relevant. The remaining covariates *x*_*j*_,*j*=61,…,1000 are considered not biologically relevant and are generated independently. In other words, we have a total of 60 biologically relevant variables. Of these, 20 were used in generating model. The remaining biologically relevant variables can therefore be considered to have zero *β* values.

We split the data into three sets each of size 100: the ‘first stage’ set $\mathcal {D}_{1}$, the ‘second stage’ set $\mathcal {D}_{2}$, and the validation set $\mathcal {D}_{3}$. We used 5-fold cross validation to select the penalty parameter in the lasso using data set $\mathcal {D}_{1}$. Bag types B1 and B3 were fixed to have size *q*=20; we used the correlation threshold 0.25 for bag type B2. We did 100 simulation runs for each experiment, that means we simulated 100 data sets using our simulation process and split into three parts $\mathcal {D}_{1}$, $\mathcal {D}_{2}$ and $\mathcal {D}_{3}$ for each such data set. We compared the performance by computing the prediction mean squared error (PMSE) in the independent dataset $\mathcal {D}_{3}$ for the derived models.

The 40 additional biologically relevant variables are positively correlated to the true ones as given in Table 1. For example, *x*_1_,*x*_2_,*x*_3_,*x*_4_,*x*_5_ are correlated to *x*_21_,*x*_22_,…,*x*_30_ with correlation 0.55. The level of correlation is similar to the correlation found between genes within a pathway in [13]. We use two levels of signal to noise ratios, namely *SNR*=0.5 and *SNR*=2. In the experiments we used one of three levels for (*β*_*j*_=0.1or0.2or0.8,for *j*=1,2,…,20) to generate response variable using (4).

**Table 1:** Correlation structure for simulation study 1

Covariates in true model	Biologically relevant variables	Correlation
*x*_1_,*x*_2_,*x*_3_,*x*_4_,*x*_5_	*x*_21_,*x*_22_,…,*x*_30_	*ρ*=0.55
*x*_6_,*x*_7_,*x*_8_,*x*_9_,*x*_10_	*x*_31_,*x*_32_,…,*x*_40_	*ρ*=0.6
*x*_11_,*x*_12_,*x*_13_,*x*_14_,*x*_15_	*x*_41_,*x*_42_,…,*x*_50_	*ρ*=0.65
*x*_16_,*x*_17_,*x*_18_,*x*_19_,*x*_20_	*x*_51_,*x*_52_,…,*x*_60_	*ρ*=0.7

In addition, we used standard lasso to select the variables in the first step, in alternative to adaptive lasso.

#### Sensitivity analysis

We experimented with various choices of thresholds and bag sizes, namely varying the correlation threshold for bag type B2 and the ‘*q*’ for bag types B1 and B3. We show the average number of selected variables in Table 2. The average number of biologically relevant variables is getting stable for *q*=20 for bag types B1 and B3 and when the correlation threshold is *ρ*=0.25 for bag type B2. Therefore we present results *q*=20 for bag types B1 and B3 and correlation threshold *ρ*=0.25 for bag type B2 in all our simulations.

**Table 2:** Average number and percentage of biologically relevant variables in the model with *S* *N* *R*=0.5 and (*β*_*j*_=0.1,*j*=1,2,…20)

Over 100 runs	Adaptive lasso	B1	B2	B3
Average number of selected variables	41	41	41	41
*q*=10 and *ρ*=0.1				
Average number of Biologically relevant variables	12	20	15	14
Average percentage of Biologically relevant variables	29.3 %	48.8 %	36.6 %	34.1 %
Standard deviation	9.1	0.86	1.24	3.11
*q*=20and *ρ*=0.25				
Average number of Biologically relevant variables	12	38	34	29
Average percentage of Biologically relevant variables	29.3 %	92.7 %	82.9 %	70.7 %
Standard deviation	9.1	0.85	1.22	3.08
*q*=30and *ρ*=0.3				
Average number of Biologically relevant variables	12	39	35	30
Average percentage of Biologically relevant variables	29.3 %	95.1 %	85.4 %	73.2 %
Standard deviation	9.1	0.85	1.23	3.09
*q*=40and *ρ*=0.4				
Average number of Biologically relevant variables	12	39	36	31
Average percentage of Biologically relevant variables	29.3 %	95.1 %	87.8 %	75.6 %
Standard deviation	9.1	0.84	1.22	3.07
Over 100 runs	Lasso	B1	B2	B3
Average number of selected variables	53	53	53	53
*q*=10 and *ρ*=0.1				
Average number of Biologically relevant variables	12	24	18	16
Average percentage of Biologically relevant variables	22.6 %	45.3 %	34.0 %	30.2 %
Standard deviation	10.2	0.99	1.55	3.94
*q*=20and *ρ*=0.25				
Average number of Biologically relevant variables	12	46	39	34
Average percentage of Biologically relevant variables (%)	22.6 %	86.8 %	72.1 %	64.2 %
Standard deviation	10.2	0.97	1.53	3.89
*q*=30and *ρ*=0.3				
Average number of Biologically relevant variables	12	47	39	36
Average percentage of Biologically relevant variables (%)	22.6 %	88.7 %	73.6 %	67.9 %
Standard deviation	10.2	0.97	1.53	3.89
*q*=40and *ρ*=0.4				
Average number of Biologically relevant variables	12	48	40	37
Average percentage of Biologically relevant variables (%)	22.6 %	90.6 %	75.5 %	69.8 %
Standard deviation	10.2	0.96	1.51	3.86

Percentage and standard deviations are over 100 runs from data $\mathcal {D}_{2}$ using correlation structure for simulation study 1 with different bag sizes *q* and correlation thresholds *ρ* from Adaptive lasso and Bag types B1, B2 and B3 based on Adaptive lasso selection and also from Lasso and Bag types B1, B2 and B3 based on lasso selection

#### Simulation results

First we investigate to what extent our replacement method generates final models with more biologically relevant variables than using adaptive lasso or standard lasso alone.

Table 3 shows that selecting bags of types B1 and B2 leads to more biologically relevant variables than when using bag type B3. Adaptive lasso selects 29.3 *%* biologically relevant variables on average over 100 simulation runs. In our 100 runs, bag types B1 and B2 produce models with on average 92.7 *%* and 82.9 *%* biologically relevant covariates respectively, while bag type B3 has 70.7 *%*. This is more than a doubling in terms of presence of biologically relevant variables.

**Table 3:** Average number and percentage of biologically relevant variables in the model with *S* *N* *R*=0.5 and (*β*_*j*_=0.1,*j*=1,2,…20)

Over 100 runs	Adaptive lasso	B1	B2	B3
Average number of selected variables	41	41	41	41
Average number of Biologically relevant variables	12	38	34	29
Average percentage of Biologically relevant variables (%)	29.3 %	92.7 %	82.9 %	70.7 %
Standard deviation	9.1	0.85	1.22	3.08
PMSE (absolute)	1.148	1.145	1.143	1.154
PRPMSE %	100 %	99.7 %	99.6 %	100.5 %
(St.dev)		(0.89)	(1.53)	(5.98)
Favorable substitution %		91 %	78 %	69 %
(St.dev)		(0.99)	(1.07)	(6.93)
MISE	1.572	1.598	1.605	1.643
Over 100 runs	Lasso	B1	B2	B3
Average number of selected variables	53	53	53	53
Average number of Biologically relevant variables	12	46	39	34
Average percentage of Biologically relevant variables (%)	22.6 %	86.8 %	72.1 %	64.2 %
Standard deviation	10.2	0.97	1.53	3.89
PMSE (absolute)	1.576	1.570	1.567	1.596
PRPMSE %	100 %	99.6 %	99.4 %	101.3 %
(St.dev)		(0.97)	(1.81)	(6.71)
Favorable substitution %		88 %	71 %	63 %
(St.dev)		(1.02)	(1.52)	(7.68)
MISE	1.876	1.914	1.927	1.941

Percentage and standard deviations are over 100 runs from data $\mathcal {D}_{2}$. The average of the PMSE and PRPMSE over 100 runs and the percentage of such runs for which the bootstrap 95 % CI includes 1 or less than 1 and mean integrated squared error (MISE), with *S* *N* *R*=0.5 and (*β*_*j*_=0.1,*j*=1,2,…,20) from data $\mathcal {D}_{3}$ using correlation structure for simulation study 1

To compare the predictive performance of using different type of bags, we report in percentage the average (over 100 runs) ratio between PMSE of the substitute model and the adaptive lasso model(PRPMSE), using data $\mathcal {D}_{3}$ for each type of bag, see Table 3. It shows that generating the model from bag types B1 and B2 performs slightly better than the adaptive lasso in terms of prediction performance — i.e. the predictive ability of the model is improved by incorporating the information on which genes are biologically relevant. But generating model from bag type B3 did not lead to improvement in terms of prediction.

To investigate this closer, we calculated the 95 *%* confidence interval for the PRPMSE, for each of the 100 simulation runs, by bootstrap. This gave 100 confidence intervals and we computed the proportion of those that contain 1 or less than 1, which would mean that the replacement does not significantly worsen the prediction power of the model. This percentage of favorable replacements is given in Table 3.

We compute the mean integrated squared error to compare the estimation accuracy of the approaches and report it in Table 3. It shows that the estimation accuracy is almost the same for all selection procedures, but our proposed bags give more biologically relevant variables than the adaptive lasso.

The results from the standard lasso is given in the bottom of Table 3. A larger number of variables are selected by adaptive lasso as expected. We get a lower percentage of biologically relevant variables compared to plain lasso. We also get a slightly lower percentage of favorable replacements from lasso than adaptive lasso. Predictive performance is worse for the lasso based models than for adaptive lasso. As before, compared with the lasso, for B1 and B2 there is an improvement in predictive performance; however, there is a deterioration in performance using bag type B3.

Other choices for the correlations than the one given in Table 1, are reported in Appendix A of the Additional file 1. They gave qualitatively similar results, see Table 2 in Appendix B in Additional file 1, where we used *SNR*=0.5 and (*β*_*j*_=0.1,*j*=1,2,…,20). Table 3 in Appendix B in Additional file 1 reports an experiment with *SNR*=0.5 and (*β*_*j*_=0.2,*j*=1,2,…,20). In Table 4 in Appendix B in Additional file 1 we used *SNR*=0.5 but (*β*_*j*_=0.8,*j*=1,2,…,20).

Next we decreased the noise level to *SNR*=2. We used (*β*_*j*_=0.1,*j*=1,2,…,20) to generate the response variables using model (4) as before. Table 4 shows that the results are very similar to the previous simulations: Bag type B1 and B2 performed better than Bag type B3, and the quality improved with reduced noise.

**Table 4:** Average number and percentage of biologically relevant variables in the model with *S* *N* *R*=2 and (*β*_*j*_=0.1,*j*=1,2,…20)

Over 100 runs	Adaptive lasso	B1	B2	B3
Average number of selected variables	44	44	44	44
Average number of Biologically relevant variables	16	42	41	37
Average percentage of Biologically relevant variables (%)	36.4 %	95.5 %	93.2 %	84.1 %
Standard deviation	9.01	0.92	1.11	3.91
PMSE (absolute)	1.895	1.878	1.880	1.901
PRPMSE %	100 %	99.1 %	99.2 %	100.3 %
(St.dev)		(1.01)	(2.14)	(6.16)
Favorable substitution %		92 %	78 %	70 %
(St.dev)		(0.99)	(1.37)	(8.92)
MISE	1.986	2.003	2.052	2.097
Over 100 runs	Lasso	B1	B2	B3
Average number of selected variables	55	55	55	55
Average number of Biologically relevant variables	16	48	45	40
Average percentage of Biologically relevant variables (%)	22.8 %	87.3 %	81.8 %	72.7 %
Standard deviation	10.2	0.99	1.53	4.12
PMSE (absolute)	2.132	2.104	2.117	2.158
PRPMSE %	100 %	98.7 %	99.3 %	101.2 %
(St.dev)		(1.11)	(2.31)	(6.89)
Favorable substitution %		89 %	70 %	68 %
(St.dev)		(1.01)	(1.69)	(9.53)
MISE	2.234	2.298	2.306	2.342

Percentage and standard deviations are over 100 runs from data $\mathcal {D}_{2}$. The average of the PMSE and PRPMSE over 100 runs and the percentage of such runs for which the bootstrap 95 % CI includes 1 or less than 1 and mean integrated squared error (MISE), with *S* *N* *R*=2 and (*β*_*j*_=0.1,*j*=1,2,…,20) from data $\mathcal {D}_{3}$ using correlation structure for simulation study 1

### Bone mineral density data

We applied our method to a gene expression data set previously studied in [14]. The data have been submitted to the European Bioinformatics Institute (EMBL-EBI) ArrayExpress repository, ID: E-MEXP-1618. R-code is given in Appendix C in Addtional file 1. For 84 women who had a trans-iliacal bone biopsy, gene expression measurements for 22815 gene probes were obtained. The data were normalized as described in [14] and we fit a linear regression model with *L*_1_ penalization with bone mineral density as response and preselected *P*=8649 covariates with the largest empirical variance.

We split the data into two sets, one set for training (2/3 of data) and the rest for validation of the selected model. We used the training data and run adaptive lasso using 5-fold cross validation. We applied our proposed method with the three different bag types. We computed predicted means square error (PMSE) using the test data for the adaptive lasso selected model and for the replaced models, to measure the percentage of loss in prediction. In Tables 5, 6 and 7 of Appendix B in Additional file 1 we list all genes selected by Lasso and their bags, for Bag type B1, B2 and B3 respectively. We used bags with 20 genes or correlation threshold 0.5. Our expert biologist (SR) selected the biologically relevant genes from each bag, and possible substitutions with the lasso genes were carried out, as detailed in the methods section. This led to new models with genes as in Table 6. The prediction in the test set was measured comparing the new models with the adaptive lasso model. Also, we re-analysed the data using a bootstrapping approach and present the averaged PMSE over 100 bootstrap samples(B-PMSE) in Table 6. Similarly, we present the list of the genes selected by standard lasso and the new models based on our bags, PMSE and B-PMSE in Table 5.

**Table 5:** Selected genes from adaptive lasso and each bag type (B1, B2, B3) based on biological reasons and PMSE and averaged PMSE over 100 bootstrap samples (B-PMSE) in the test data

	Adaptive lasso	Bag type B1	Bag type B2	Bag type B3
	AK3L1	ADAMTS2	ADAMTS2	RARA
	CSRNP3	RUNX2	RUNX2	CSRNP3
	FKBP14	SPTBN1	SPTBN1	VDR
	NF1	NF1	NF1	CSNK1G3
	PIAS4	ESR1	ESR1	PIAS4
	PLIN5	PLIN5	HGF	RHO
	PPIL2	BMP5	BMP5	SRGAP3
	RNF31	RNF31	RNF31	GLP1R G
	SRR	SMAD3	SRR	SRR
	TRPS1	SFRP1	SFRP1	TRPS1
	ZMAT3	ZMAT3	ZMAT3	ZMAT3
PMSE	1.292	0.251	0.343	1.306
B-PMSE	1.074	0.242	0.298	1.286

**Table 6:** Selected genes from lasso and each bag type (B1, B2, B3) based on biological reasons and PMSE and averaged PMSE over 100 bootstrap samples (B-PMSE) in the test data

	Lasso	Bag type B1	Bag type B2	Bag type B3
	AK3L1	ADAMTS2	ADAMTS2	RARA
	CCHCR1	CCHCR1	CCHCR1	CCHCR1
	CRYGS	PPARA	PPARA	ESR1
	CSRNP3	RUNX2	RUNX2	CSRNP3
	FAF1	FAF1	FAF1	BMPR2
	FKBP14	SPTBN1	SPTBN1	VDR
	FLRT2	PDGFA	PDGFA	FLRT2
	KDM4A	SLC44A1	KDM4A7	KDM4A
	LOC642852	OSTM1	OSTM1	LOC642852
	MAPK8	BMP7	BMP7	WHAMML1 /// WHAMML2
	NF1	NF1	NF1	CSNK1G3
	PIAS4	ESR1	ESR1	PIAS4
	PLIN5	PLIN5	HGF	RHO
	PPIL2	BMP5	BMP5	SRGAP3
	RNF31	RNF31	RNF31	GLP1R G
	SRR	SMAD3	SRR	SRR
	TRPS1	SFRP1	SFRP1	TRPS1
	ZMAT3	ZMAT3	ZMAT3	ZMAT3
PMSE	1.900	1.009	1.086	5.810
B-PMSE	1.871	1.001	1.024	5.053

We can see from Tables 5 and 6, that the PMSE is smaller for the models based on bag types B1 and B2 compared with the initial adaptive lasso or lasso method. This demonstrates the merits of the proposed method: the model with more biologically relevant genes also leads to better out of sample predictions. On the other hand, the PMSE for bag type B3 is larger than for the other methods.

## Discussion

We have presented a method that allows biological relevance of a large number of covariates to be elicited in a feasible manner, by fitting an initial model to generate a list of ‘interesting’ covariates. This list is produced by using an adaptive lasso model to generate a shortlist of variables, then extending this list to include a ‘bag’ of variables that are related to the original variables. Each gene in the union of these bags can then be classed, by a domain expert, as biologically interesting or not, and the adaptive lasso variables can be substituted for biologically interesting genes where possible. Simulations and a real data example demonstrate that our method leads to a model with more biological relevance, and better out-of-sample predictions compared with applying adaptive lasso alone, and we found similar results using standard lasso.

Based on our simulations, we recommend populating the ‘bags’ using genes highly correlated with the adaptive lasso genes, rather than considering the PMSE. We recommend a correlation threshold of 0.25 for constructing these bags; however, it is useful to conduct sensitivity analysis around this threshold to ensure that there is stability in selection of biologically relevant variables.

It is of interest to explore why bag types 1 and 2 (correlation based) perform better than bag type 3 (PMSE based). Our initial investigations show that this is because, with the PMSE bags, a small number of biologically relevant genes appear in many bags. This leads to a number of duplicate substitutions, and hence a sparser final model.

A strength of this approach is that it is more efficient to perform than a fully informative Bayesian approach, which would involve eliciting priors scaled to all genes. However, it takes longer than applying adaptive lasso or lasso with no incorporation of prior information. We believe the approach is a good trade-off between modelling effort, domain expert input, and quality of the final model. The involvement of domain experts in the modelling process is also a strength because they may perceive the resulting model as being more relevant as they have invested in its production. A weakness is that the ‘pre screening’ stage could eliminate genes that the domain experts have a very strong prior belief of involvement in the relevant mechanistic processes. This could be alleviated by allowing a small number of prior known genes to appear in the substitution bags regardless of meeting the correlation or PMSE criteria. For clarity, we explained the method using linear models, but the extension to generalised linear models and multilevel models is clear. One possible criticism of the approach is that the preference for using ‘known’ genes in the final models may prevent discovery of novel mechanisms. If there is a very strong signal for an unknown gene being a prognosticator, this would survive the post-processing to appear in the final model.

This work could be extended by incorporating richer biological information about the genes — we used a binary classification of ‘interesting’ and ‘not interesting’ — this could be extended to a continuous importance measure of each gene. The challenge then would be how to weight the biological information appropriately against the predictive ability.

## Conclusion

Our method allows feasible incorporation of biological knowledge into predictive models with a large number of potential covariates, leading to models with greater face validity, generalisability and interpretability, without adversely affecting predictive performance.

## Additional file

Additional file 1 More simulation results and interpretations in Appendix A and Appendix B. R-code for data is presented in Appendix C. (PDF 141 kb)
