# Supplementary material for: Tilting the lasso by knowledge-based post-processing
Source: BMC Bioinformatics. 2016 Sep 2;17(1):344. doi: 10.1186/s12859-016-1210-7 (PMC5010709; doi:10.1186/s12859-016-1210-7)
Supplement: Additional file 1 — More simulation results and interpretations in Appendix A and Appendix B. R-code for data is presented in Appendix C. (PDF 141 kb) [file 12859_2016_1210_MOESM1_ESM.pdf]

Supplementary Materials for Tilting the Lasso by Knowledge Based Post Processing

Kukatharmini Tharmaratnam , Matt Sperrin, Thomas Jaki, Sjur Reppe and Arnoldo Frigessi

The supplementary material contains Experiment 2 for correlation structure of the covariates and four tables with results from different simulation experiments.

1 Appendix A

In this case correlations are less than the correlation structure for simulation study 1.

Table 1: Correlation structure for simulation study 2

Covariates in True model	Biologically relevant variables	correlation
x_1, x_2, x_3, x_4, x_5	$x_{21}, x_{22}, \ldots, x_{30}$	$\rho = 0.5$
$x_6, x_7, x_8, x_9, x_{10}$	$x_{31}, x_{32}, \ldots, x_{40}$	$\rho = 0.4$
$x_{11}, x_{12}, x_{13}, x_{14}, x_{15}$	$x_{41}, x_{42}, \ldots, x_{50}$	$\rho = 0.35$
$x_{16}, x_{17}, x_{18}, x_{19}, x_{20}$	$x_{51}, x_{52}, \ldots, x_{60}$	$\rho = 0.3$

2 Appendix B

We generate covariates with a $SNR = 0.5$ and ($\beta_j = 0.1, j = 1, 2, \ldots, 20$) to generate response variables using model (3) and correlation structure for simulation study 2 as in Table1. We investigate to what extend our replacement method allows to propose models with more biologically relevant variables.

Table 2 shows that selecting bags with methods B1 and B2 select more biologically relevant variables than bag type B3 method. Lasso select only 15% biologically relevant covariates from 100 simulation runs. Our proposed bag type B1 selects around 85% biologically relevant covariates, while other bag types B2 and B3 select 73% and 67% respectively. Table 2 gives similar results to the correlation structure for simulation study 1.

To compare the performance of different type of bags, we computed percentage of ratio between PMSE for each type of bag (B1, B2 and B3) for 100 simulation runs. The average of PRPMSE is given in Table 2. It shows that generating the model from bag types B1 and B2 perform better than bag type B3. We calculated the 95% confidence interval for

Table 2: Average number and percentage of biologically relevant variables in the model with $SNR = 0.5$ and ($\beta_j = 0.1, j = 1, 2, \ldots 20$). Percentage and standard deviations are over 100 runs from data $\mathcal{D}_2$. The average of the PRPMSE over 100 runs and the percentage of such runs for which the bootstrap 95% CI includes 1 or less than 1, with $SNR = 0.5$ and ($\beta_j = 0.1, j = 1, 2, \ldots, 20$) from data $\mathcal{D}_3$ using correlation structure for simulation study 2

Over 100 runs	Adaptive Lasso	B1	B2	B3
Average number of selected variables	39	39	39	39
Average number of Biologically relevant variables	8	35	32	30
Averageof Biologically relevant variables (%)	20.5%	89.7%	82.1%	76.9%
Standard deviation	9.8	1.02	1.69	4.38
PMSE (absolute)	1.289	1.272	1.262	1.304
PRPMSE %	100%	98.7%	97.9%	101.2%
(St.dev)		(1.05)	(1.29)	(3.12)
Favorable substitution%		87%	72%	66%
(St.dev)		(1.01)	(0.92)	(4.34)
MISE	1.687	1.699	1.731	1.782
Over 100 runs	Lasso	B1	B2	B3
Average number of selected variables	52	52	52	52
Average number of Biologically relevant variables	8	44	38	35
Averageof Biologically relevant variables (%)	14.8%	84.6%	73.1%	67.3%
Standard deviation	11.1	1.46	2.09	5.81
PMSE (absolute)	1.697	1.665	1.656	1.733
PRPMSE %	100%	98.1%	97.6%	102.1%
(St.dev)		(1.11)	(1.41)	(3.66)
Favorable substitution%		84%	69%	61%
(St.dev)		(1.06)	(0.97)	(5.01)
MISE	1.995	2.049	2.076	2.983

the percentage of ratio between PMSE (PRPMSE) for each of the 100 simulation runs by bootstrap as described in the main manuscript. Table 2 shows that bag types B1 and B2 are selecting the biologically relevant covariates around 85% and 70% favorable replacement respectively. Bag type B3 selects around 60% favorable substitution.

The results from the adaptive lasso is given in the bottom of Table 2. It shows that less number of variable selected from adaptive lasso as we expected. We get higher percentage of biologically relevant variables on average over 100 simulation runs than lasso selection case. We also get slightly higher percentage of favorable replacements from adaptive lasso than lasso.

We present mean integrated square error in Table 2 and it shows that, MISE are slightly lager than lasso selected variables with our proposed bag types variables. But we can get more boilogically relevant variables from our proposed bag types. We can see simillar results with adaptive lasso as well.

We use $SNR = 0.5$ and ($\beta_j = 0.2, j = 1, 2, \ldots, 20$) values for 20 variables $x_j, j = 1, 2, \ldots, 20$ to generate response variable using (3) and experiment 1 as described in the

main manuscript. Table 3 shows that selecting bags with methods B1 and B2 selects

Table 3: Average number and percentage of biologically relevant variables in the model with $SNR = 0.5$ and ($\beta_j = 0.2, j = 1, 2, \ldots 20$). Percentage and standard deviations are over 100 runs from data $\mathcal{D}_2$. The average of the PRPMSE over 100 runs and the percentage of such runs for which the bootstrap 95% CI includes 1 or less than 1, with $SNR = 0.5$ and ($\beta_j = 0.2, j = 1, 2, \ldots, 20$) from data $\mathcal{D}_3$ using correlation structure for simulation study 1

Over 100 runs	Adaptive Lasso	B1	B2	B3
Average number of selected variables	40	40	40	40
Average number of Biologically relevant variables	13	38	35	31
Averageof Biologically relevant variables (%)	32.5%	95.0%	87.5%	77.5%
Standard deviation	9.73	0.89	1.24	3.89
PMSE (absolute)	1.102	1.099	1.095	1.116
PRPMSE %	100%	99.7%	99.4%	101.3%
(St.dev)		(1.34)	(2.15)	(6.72)
Favorable substitution%		89%	71%	69%
(St.dev)		(1.67)	(2.07)	(7.54)
MISE	2.432	2.486	2.519	2.538
Over 100 runs	Lasso	B1	B2	B3
Average number of selected variables	54	54	54	54
Average number of Biologically relevant variables	13	47	43	37
Averageof Biologically relevant variables (%)	23.4%	87.0%	79.1%	68.5%
Standard deviation	10.8	0.96	1.80	4.56
PMSE (absolute)	1.528	1.522	1.514	1.572
PRPMSE %	100%	99.6%	99.1%	102.9%
(St.dev)		(1.62)	(2.82)	(7.56)
Favorable substitution%		85%	67%	64%
(St.dev)		(2.13)	(2.52)	(8.12)
MISE	2.867	2.906	2.954	2.981

more biologically relevant variables than B3. To compare the performance of different type of bags, we computed PRPMSE for each type of bag (B1, B2 and B3) and lasso selected model for 100 simulation runs. The average of the PRPMSE is given in Table 3. It shows that generating the model from bag types B1 and B2 perform better than bag type B3 in terms of prediction performance.

We use $SNR = 0.5$ and ($\beta_j = 0.8, j = 1, 2, \ldots, 20$) values for 20 variables $x_j, j = 1, 2, \ldots, 20$ to generate response variable using (3) and experiment 1 as described in the main manuscript. Table 4 shows that selecting bags with methods B1 and B2 selects more biologically relevant variables than B3. To compare the performance of different type of bags, we computed PRPMSE for each type of bag (B1, B2 and B3) and lasso selected model for 100 simulation runs. The average of the PRPMSE is given in Table 4. It shows that generating the model from bag types B1 and B2 perform better than bag type B3 in terms of prediction performance.

We calculated the 95% confidence interval for the PRPMSE for each 100 simulation

Table 4: Average number and percentage of biologically relevant variables in the model with $SNR = 0.5$ and ($\beta_j = 0.8, j = 1, 2, \ldots 20$). Percentage and standard deviations are over 100 runs from data $\mathcal{D}_2$. The average of the PRPMSE over 100 runs and the percentage of such runs for which the bootstrap 95% CI includes 1 or less than 1, with $SNR = 0.5$ and ($\beta_j = 0.8, j = 1, 2, \ldots, 20$) from data $\mathcal{D}_3$ using correlation structure for simulation study 1

Over 100 runs	Adaptive Lasso	B1	B2	B3
Average number of selected variables	42	42	42	42
Average number of Biologically relevant variables	13	39	36	32
Averageof Biologically relevant variables (%)	31.0%	92.9%	85.7%	76.2%
Standard deviation	9.54	0.82	1.19	3.58
PMSE (absolute)	1.143	1.140	1.134	1.164
PRPMSE %	100%	99.7%	99.2%	101.8%
(St.dev)		(1.29)	(2.01)	(6.46)
Favorable substitution%		88%	70%	67%
(St.dev)		(1.73)	(2.16)	(7.41)
MISE	2.583	2.589	2.594	2.608
Over 100 runs	Lasso	B1	B2	B3
Average number of selected variables	56	56	56	56
Average number of Biologically relevant variables	13	48	44	38
Averageof Biologically relevant variables (%)	23.2%	85.7%	78.6%	67.9%
Standard deviation	11.9	0.99	1.860	5.04
PMSE (absolute)	1.485	1.482	1.475	1.522
PRPMSE %	100%	99.8%	99.3%	102.5%
(St.dev)		(1.51)	(2.67)	(7.03)
Favorable substitution%		87%	69%	62%
(St.dev)		(2.00)	(2.23)	(7.62)
MISE	2.916	2.935	2.973	2.996

runs by bootstrap as described in the main manuscript. Table 3 confirms that bag types B1 and B2 performs better than B3. The adaptive lasso results show simillar patteren as well.

Table 5: Selection of genes from lasso and corresponding bags from bag type B1, percentage of ratio between PMSE

Genes	Genes in the Bags									
from Lasso	1	2	3	4	5	6	7	8	9	10
AK3L1	FLJ39051	ADAMTS2	RRP12	PSMB2	SUN5	PKLR	SFN	RBL1	C19orf6	PGAP3
PRPMSE %	157	99	163	163	161	99	174	169	154	154
CCHCR1	CCHCR1	KLHL22	STARD10	SERHL2	B3GNT6	KBTBD4	DGCR14	LACTB	EHD1	ERCC6
PRPMSE%	115	108	105	129	107	113	141	110	123	104
CRYGS	LACTB	CD5	FAM115A	SEC16B	USP46	CRIM1	SYNRG	PPARA	CPEB3	UBR5
PRPMSE %	69	88	69	70	70	74	63	94	68	57
CSRNP3	RUNX2	UNC5B	UNC13D	BMP2	FAM20C	TRIM2	CDK13	MMP16	ZFHX4	BMP7
PRPMSE %	158	120	133	125	126	124	122	98	145	88
FAF1	CAMSAP1	ZNF75A	EXOG	RFFL	ABLIM2	ARPC5L	CRCP	EIF5B	NLGN2	TNRC18
PRPMSE %	129	118	103	102	90	119	114	103	118	113
FKBP14	C12orf47	SS18	TACC1	SPAG9	THBD	GTF2H2B	FNDC5	CRYGS	ZBTB43	SPTBN1
PRPMSE %	91	224	227	151	206	182	189	171	112	228
FLRT2	PDGFA	EDNRA	ITGBL1	LMO7	ITGBL1	NRP2	LOC728190	MYO6	LAMA4	SEPT11
PRPMSE %	63	55	86	79	57	57	63	54	45	64
KDM4A	SLC44A1	FUT7	ING3	GRB10	TBX2	PPIL2	SDCCAG8	CPPED1	PPARA	EIF5B
PRPMSE %	107	117	104	113	108	118	101	126	124	77
LOC642852	ADAMTSL1	PRKAG2	CEP164	ABAT	ZNF45	OGFRL1	CTU2	THEM4	SARM1	ATP5S
PRPMSE %	88	105	80	75	66	88	85	72	82	111
MAPK8	PFKFB2	RIT1	CASZ1	F7	BMP7	SLA2	FTCD	CHDH	SNAPC4	CAMK2D
PRPMSE %	97	91	157	111	99	99	91	73	98	112
NF1	MYH14	ZNF20 /// ZNF625	ASXL3	LTBP4	KLK4	ZFHX4	FOXN4	TLE4	GTF3C4	IL17RC
PRPMSE %	145	142	124	116	150	144	156	139	129	163
PIAS4	GFPT1	CEP152	SH3RF1	SP3	B3GNT6	DHX30	TTC28	TCEA2	PLAC2	DIO2
PRPMSE %	38	45	48	59	68	46	44	52	75	53
PLIN5	NOX1	SFTPC	MESP2	MUC5AC	SETDB2	NPR3	IL1RL1	PROM2	VAV2	TMEM 161B
PRPMSE %	80	83	92	92	75	88	115	98	86	56
PPIL2	ACTG1	ACTG1	BMP5	ARSK	ITGBL1	C9orf167	PCIF1	KLHL18	ITGBL1	SIN3A
PRPMSE %	108	105	118	103	101	106	115	104	104	91
RNF31	SEPT8	SENP2	CMAH	B3GAT3	MLLT10	KIAA0415	FLJ44342	GFPT1	BCL3	GALNT5
PRPMSE %	97	94	96	130	95	100	99	82	94	100
SRR	C1orf163	FAM38B	TNFRSF10A	ERCC6	THBD	PDCD2	SMAD3	RRN3P1	EGFR	PDE4A
PRPMSE %	53	67	58	73	55	47	76	60	61	56
TRPS1	FLRT2	LMO7	WWC2	EDNRA	ITPRIPL2	PDGFA	ZHX3	TRPC6	SFRP1	SEPT11
PRPMSE %	NA	187	192	199	157	176	205	202	114	161
ZMAT3	CNOT3	MARK4	WDR74	ERCC1	HSPA1L	FBXO9	WDR27	FOSL2	GOSR2	C15orf39
PRPMSE %	155	150	150	160	146	152	106	153	157	169

(/// between two genes indicates that transcripts from both genes are detected by the relevant Affymetrix probe set)

Table 6: Selection of genes from lasso and corresponding bags from bag type B2 (correlation> 0.55), percentage of ratio between PMSE

Genes from Lasso	Genes in the Bags									
AK3L1	FLJ39051	ADAMTS2								
PRPMSE %	157	99								
CCHCR1	CCHCR1	KLHL22	STARD10							
PRPMSE %	115	108	105							
CRYGS	LACTB	CD5	FAM115A	SEC16B	USP46	CRIM1	SYNRG	PPARA	CPEB3	UBR5
PRPMSE %	69	88	69	70	70	74	63	94	68	57
CRYGS	NAA25	TARSL2	EPHB4	FANCA	C7orf50	GNAS	PEX13	ZNF385B		
PRPMSE %	70	89	75	109	94	75	67	64		
CSRNP3	RUNX2	UNC5B	UNC13D	BMP2	FAM20C	TRIM2	CDK13	MMP16	ZFHX4	
PRPMSE %	158	120	133	125	126	124	122	98	145	
FAF1	CAMSAP1	ZNF75A	EXOG	RFFL	ABLIM2	ARPC5L	CRCP	EIF5B	NLGN2	TNRC18
PRPMSE %	129	118	103	102	90	119	114	103	118	113
FKBP14	C12orf47	SS18	TACC1	SPAG9						
PRPMSE %	91	224	227	151						
FLRT2	PDGFA	EDNRA	ITGBL1	LMO7	ITGBL1	NRP2	LOC728190	MYO6	LAMA4	SEPT11
PRPMSE %	63	55	86	79	57	57	63	54	45	64
FLRT2	TRPS1	TMTC2	PTPRG	COL11A1	SSH3	PCIF1	CHAF1A	ZNF341	UNC5B	SMAD4
PRPMSE %	60	50	60	56	57	55	55	65	57	90
FLRT2	ITGB4									
PRPMSE %	78									
KDM4A										
LOC642852	ADAMTSL1	PRKAG2	CEP164							
PRPMSE %	88	105	80							
MAPK8	PFKFB2									
PRPMSE %	97									
NF1										
PIAS4	GFPT1	CEP152								
PRPMSE %	38	45								
PLIN5	NOX1	SFTPC	MESP2	MUC5AC	SETDB2	NPR3	IL1RL1	PROM2	VAV2	TMEM161B
PRPMSE %	80	83	92	92	75	88	115	98	86	56
PLIN5	ABHD11	NFYC	ZNF780A	FLJ38717	ADPRHL1	CELF4				
PRPMSE %	87	87	85	92	44	87				
PPIL2	ACTG1	ACTG1	BMP5	ARSK	ITGBL1					
PRPMSE %	108	105	118	103	101					
RNF31	SEPT8	SENP2	CMAH	B3GAT3	MLLT10	KIAA0415	FLJ44342	GFPT1	BCL3	
PRPMSE %	97	94	96	130	95	100	99	82	94	
SRR	C1orf163									
PRPMSE %	53									
TRPS1	FLRT2	LMO7	WWC2							
PRPMSE %	NA	187	192							
ZMAT3										

Some genes are denoted as follows,(SAA1 /// SAA2)=SAA1/2 , (ZNF20 /// ZNF625)=ZNF20/625, (D4S234E /// FOXP1)=D4S234E1, RAB11FIP4 =RAB1 (/// between two genes indicates that transcripts from both genes are detected by the relevant Affymetrix probe set)

Table 7: Selection of genes from lasso and corresponding bags from bag type B3, percentage of ratio between PMSE

Genes from Lasso	Genes in the Bags 1	2	3	4	5	6	7	8	9	10
AK3L1	WWP2	ETV5	SRGAP3	ECE2	SRCIN1	RARA	MFI2	CLDN11	OSBPL10	HPX
PRPMSE %	147	130	231	156	180	173	163	175	119	109
CCHCR1	GLP1R	SRGAP3	ECE2	KCTD1	WHA1/2	IL28RA	HPX	CSNK	C16o	NTRK3
PRPMSE %	124	140	119	121	120	132	93	136	104	99
CRYGS	ECE2	WHA1/2	NTRK3	SRGAP3	HPX	IL28RA	GLP1R	KCTD1	CSNK	WWP2
PRPMSE %	65	71	58	79	56	100	70	76	77	72
CSRNP3	GLP1R	ECE2	CSNK	KCTD1	HFE	C15orf42	NTRK3	INF2	NUP188	EHMT2
PRPMSE %	152	139	160	160	107	136	125	104	137	104
FAF1	ECE2	CSNK	KCTD1	BMPR2	CROCC	WHA1/2	PSAT1	NCLN	RNF157	GLP1R
PRPMSE %	118	141	147	142	66	122	117	135	75	132
FKBP14	SRGAP3	IGF2BP1	ECE2	WWP2	KCTD1	PPP2R3B	C16o	LOC4	TUB	SRCIN1
PRPMSE %	252	235	195	186	237	190	182	172	135	204
FLRT2	GLP1R	ECE2	WHA1/2	CSNK	HPX	MFI2	NTRK3	IL28RA	C16o	ZNF561
PRPMSE %	69	58	64	84	38	64	50	71	53	65
KDM4A	GLP1R	ECE2	SRGAP3	HPX	MFI2	IL28RA	KCTD1	WHA1/2	NTRK3	CSNK
PRPMSE %	128	115	149	96	124	144	139	117	105	132
LOC642852	GLP1R	SRGAP3	ECE2	IL1RL1	PRKAR1B	TRPC4	CLDN19	C7orf63	NLGN2	CADM3
PRPMSE %	108	127	90	110	69	63	91	78	107	108
MAPK8	WHA1/2 WHAMML2	CSNK 1G3	ECE2	C16o	MFI2	ALG10B	WWP2	LOC3 39290	HPX	ZNF780A
PRPMSE %	107	131	103	96	112	124	99	108	81	94
NF1	CSNK	C15orf42	PSAT1	WHA1/2	FUT6	HNF1B	GLP1R	C21orf2	TRPC6	KCTD1
PRPMSE %	173	136	144	151	143	129	168	162	140	184
PIAS4	WHA1/2	TRPC4	FCER2	CSNK	BICD1	NTRK3	ST7L	LOC4	ALG10B	RECQL5
PRPMSE %	58	41	58	69	60	41	41	37	70	58
PLIN5	GLP1R	IL28RA	C16o	IL1RL1	NTRK3	PPP2R3B	NCS1	HNF1B	WWP2	FUT6
PRPMSE %	116	135	95	115	86	101	73	89	93	104
PPIL2	SRGAP3	ECE2	HPX	GLP1R	MFI2	WHA1/2	KCTD1	IL28RA	CLDN11	WWP2
PRPMSE %	140	109	84	123	113	111	135	139	116	104
RNF31	GLP1R	SRGAP3	KCTD1	ECE2	HPX	NTRK3	IL28RA	CLDN11	FCRL2	C16o
PRPMSE %	111	129	128	93	81	89	135	111	104	99
SRR	GLP1R	ECE2	WHA1/2	WWP2	NTRK3	SLC25A17	C16o	MFI2	FCER2	HPX
PRPMSE %	93	61	66	63	61	114	58	77	68	45
TRPS1	GLP1R	SRGAP3	KCTD1	IL1RL1	HPX	WWP2	C21orf2	SRCIN1	ECE2	C16o
PRPMSE %	195	222	212	193	117	185	182	192	200	183
ZMAT3	GLP1R	SRGAP3	ECE2	WHA1/2	KCTD1	MFI2	NTRK3	HPX	CSNK	WWP2
PRPMSE %	184	201	172	170	195	178	154	154	190	157

Some genes are denoted as follows, (WHAMML1/// WHAMML2)= WHA1/2, C16orf45=C16o, CSNK1G3=CSNK, LOC401320= LOC4 (/// between two genes indicates that transcripts from both genes are detected by the relevant Affymetrix probe set)

3 Appendix C

```
#code for running the bone example for the paper

#packages
library(glmnet)
library(MASS)
#read in the data
bone<-read.csv("boneT.csv") ## Need to specify the path of the directry, where data stored
dim(bone)
#split data: one set for training data + aother set for test data to do validation in lasso
#THIS SECTION splits the data in 1/3 and uses 2/3 of the data used as training data

set.seed(1111)
n<-dim(bone)[1]
TRS<-sample(1:n,floor(2*n/3))
bone1<-bone[TRS,] # Training data
bone2<-bone[-TRS,] # Test data
boneX1<-as.matrix(bone[TRS,4:8652]) #predictors
boneY1<-as.vector(bone[TRS,3])  #response (Z-score)
P=dim(boneX1)[2]
nTR=dim(boneX1)[1]
colnames(boneX1)=1:P

#### Lasso fit for Training data
TRfit<-cv.glmnet(boneX1,boneY1,nfolds=5) #initial fit
plot(TRfit) #a well-defined minimum in the MSE
which.min(TRfit$lambda)
lassofit=glmnet(boneX1,boneY1,family="gaussian",lambda=TRfit$lambda.min,standardize=T)
coef=as.vector(lassofit$beta)
coef1=cbind(colnames(boneX1),coef)
nonzeroX=coef1[coef!=0][1:lassofit$df]
nonzeroX

###Make bags with respect to correlation
corrmatrixW=matrix(NA,ncol=length(nonzeroX),nrow=(P-1))
#### select 20 highly correlated covariates
absmcororderall=matrix(NA,ncol=length(nonzeroX),nrow=(P-1))
absmcororder=matrix(NA,ncol=length(nonzeroX),nrow=20)
#### select covariates with corr > 0.5
absmcorgreaterthan05 =list()
selectabsmcorgreaterthan05 =list()
 for( j in 1:length(nonzeroX)){
corrmatrixW[,j]=abs(cor(boneX1[,as.numeric(nonzeroX[j])],
                            boneX1[,-as.numeric(nonzeroX[j])]))
absmcororder[,j]=order(corrmatrixW[,j],decreasing=TRUE)[1:20] #indices of top 20 variables
for(i in 1:(P-1)){
if(corrmatrixW[i,j]>0.5) absmcororderall[i,j]= corrmatrixW[i,j]
}
```

```
absmcorgreaterthan05[j]=list(order(absmcororderall[,j],na.last = NA,decreasing=TRUE))
}

#####Fit OLS regression with selected variables from lasso and compute MSE
lsfitlasso=lm(boneY1~-1+boneX1[,c(as.numeric(nonzeroX))])
betalasso=as.matrix(lsfitlasso$coef,ncol=1,nrow=length(nonzeroX))
yhat=(boneX1[,c(as.numeric(nonzeroX))]%*%betalasso)
lsfitlassoMSE=1/nTR*sum(boneY1-yhat)^2
lsfitlassoMSE

lsMSE=matrix(NA,ncol=length(nonzeroX),nrow=P)
lsMSEdiff=matrix(NA,ncol=length(nonzeroX),nrow=P)
colname=matrix(1:P,ncol=1,nrow=P)
lsMSEdifforder=matrix(NA,ncol=length(nonzeroX),nrow=20)
for(j in 1:length(nonzeroX)){
for(i in 1:P){
newX=boneX1[,replace(as.numeric(nonzeroX),j,as.numeric(colname[i]))]
lsfitX1=lm(boneY1~-1+newX)
betalassoX1=as.matrix(c(lsfitX1$coef),ncol=1,nrow=length(nonzeroX))
yhat=(newX%*%betalassoX1)
lsMSE[i,j]=1/nTR*sum(boneY1-yhat)^2
lsMSEdiff[i,j]=((lsMSE[i,j]/lsfitlassoMSE)*100)
}
lsMSEdifforder[,j]=order(lsMSEdiff[,j],decreasing=TRUE)[1:20] #indices of top 20 variables
}

nonzeroX
absmcororder    ###Bag with 20 highly correlated variables
absmcorgreaterthan05 ###Bag with variables larger than 0.5
lsMSEdifforder                   ### Bag with 20 small MSE

##### Identify genes with respect to renamed selected covariates
genename=read.csv("C:/Users/tharmark/Desktop/Bags Project/Arnoldo project
                                       /genename.csv",sep = "\t")
dim(genename)
nonzeroXgene=genename[c(as.numeric(nonzeroX)),3]
nonzeroXgene

absmcorordergene=matrix(0,ncol=length(nonzeroX),nrow=20)
absmcorgreaterthan05gene=list()
lsMSEdiffordergene=matrix(0,ncol=length(nonzeroX),nrow=20)
for(j in 1:length(nonzeroX)){
absmcorordergene[,j]=as.matrix((genename[c(absmcororder[,j]),2]))
absmcorgreaterthan05gene[[j]]=list(genename[c(absmcorgreaterthan05[[j]]),2])
lsMSEdiffordergene[,j]=as.matrix(genename[c(lsMSEdifforder[,j]),2])
}
t(absmcorordergene)
absmcorgreaterthan05gene
t(lsMSEdiffordergene)
```

```
##### prediction mean squares error ######
testn=n-nTR
boneX2<-as.matrix(bone[-TRS,4:8652]) #predictors
boneY2<-as.vector(bone[-TRS,3])  #response (Z-score)

nonzeroX
Lasso.yhat=boneX2[,c(as.numeric(nonzeroX))]%*%betalasso  ##### using test data
Lasso.PMSE= 1/testn * sum(boneY2-Lasso.yhat)^2
Lasso.PMSE

Lasso.yhat1=boneX1[,c(as.numeric(nonzeroX))]%*%betalasso  ######using training data
Lasso.MSE= 1/nTR * sum(boneY1-Lasso.yhat1)^2
Lasso.MSE

###########################
########Compute PMSE from the model with green genes #########
greengeneX1B1=boneX1[,replace(as.numeric(nonzeroX),c(1:18),c(137,1364,5735,6366,2498,
                      7066,5440,6840,5284,754,4995,2432,5449,749,6261,6881,6565,8357))]
greengeneX1B2=boneX1[,replace(as.numeric(nonzeroX),c(1:18),c(137,1364,5735,6366,2498,
                     7066,5440,3701,5284,754,4995,2432,3565,749,6261,7105,6565,8357))]
greengeneX1B3=boneX1[,replace(as.numeric(nonzeroX),c(1:18),c(6054,1364,2433,1807,760,
                     8071,2768,3701,4160,4377,1798,5562,6194,5756,6261,7105,7789,8357))]

####Bag type B1 #####
lsfitX1=lm(boneY1~-1+greengeneX1B1)
betalassoX1B1=as.matrix(c(lsfitX1$coef),ncol=1,nrow=length(nonzeroX))
newX2=boneX2[,replace(as.numeric(nonzeroX),c(1:18),c(137,1364,5735,6366,2498,
          7066,5440,6840,5284,754,4995,2432,5449,749,6261,6881,6565,8357))]
yhat2=(newX2%*%betalassoX1B1)
lsPMSEgreengeneX1=1/testn*sum(boneY2-yhat2)^2  ##### using test data
lsPMSEgreengeneX1

yhat1=(greengeneX1B1%*%betalassoX1B1) ######using training data
lsMSEgreengeneX1=1/nTR*sum(boneY1-yhat1)^2
lsMSEgreengeneX1

####Bag type B2 #####
lsfitX1=lm(boneY1~-1+greengeneX1B2)
betalassoX1B2=as.matrix(c(lsfitX1$coef),ncol=1,nrow=length(nonzeroX))
newX2=boneX2[,replace(as.numeric(nonzeroX),c(1:18),c(137,1364,5735,6366,2498,
            7066,5440,3701,5284,754,4995,2432,3565,749,6261,7105,6565,8357))]
yhat2=(newX2%*%betalassoX1B2)
lsPMSEgreengeneX1=1/testn*sum(boneY2-yhat2)^2   ##### using test data
lsPMSEgreengeneX1

yhat1=(greengeneX1B2%*%betalassoX1B2) ######using training data
lsMSEgreengeneX1=1/nTR*sum(boneY1-yhat1)^2
lsMSEgreengeneX1
```

```
####Bag type B3 #####
lsfitX1=lm(boneY1~-1+greengeneX1B3)
betalassoX1B3=as.matrix(c(lsfitX1$coef),ncol=1,nrow=length(nonzeroX))
newX2=boneX2[,replace(as.numeric(nonzeroX),c(1:18),c(6054,1364,2433,1807,760,
              8071,2768,3701,4160,4377,1798,5562,6194,5756,6261,7105,7789,8357))]
yhat2=(newX2%*%betalassoX1B3)
lsPMSEgreengeneX1=1/testn*sum(boneY2-yhat2)^2  ##### using test data
lsPMSEgreengeneX1

yhat1=(greengeneX1B3%*%betalassoX1B3) ######using training data
lsMSEgreengeneX1=1/nTR*sum(boneY1-yhat1)^2
lsMSEgreengeneX1
```
